# Lost and Found: *Coffea stenophylla* and *C. affinis*, the Forgotten Coffee Crop Species of West Africa

**DOI:** 10.3389/fpls.2020.00616

**Published:** 2020-05-19

**Authors:** Aaron P. Davis, Roberta Gargiulo, Michael F. Fay, Daniel Sarmu, Jeremy Haggar

**Affiliations:** ^1^Royal Botanic Gardens, Kew, Richmond, United Kingdom; ^2^Welthungerhilfe, Freetown, Sierra Leone; ^3^Department of Agriculture, Health and Environment, Faculty of Engineering and Science, Natural Resources Institute, University of Greenwich, Medway, United Kingdom

**Keywords:** agronomy, climate change, coffee, West Africa, crop wild relatives (CWRs), DNA, Sierra Leone, speciality coffee

## Abstract

*Coffea arabica* (Arabica) and *C. canephora* (robusta) almost entirely dominate global coffee production. Various challenges at the production (farm) level, including the increasing prevalence and severity of disease and pests and climate change, indicate that the coffee crop portfolio needs to be substantially diversified in order to ensure resilience and sustainability. In this study, we use a multidisciplinary approach (herbarium and literature review, fieldwork and DNA sequencing) to elucidate the identity, whereabouts, and potential attributes, of two poorly known coffee crop species: *C. affinis* and *C. stenophylla*. We show that despite widespread (albeit small-scale) use as a coffee crop species across Upper West Africa and further afield more than 100 years ago, these species are now extremely rare in the wild and are not being farmed. Fieldwork enabled us to rediscover *C. stenophylla* in Sierra Leone, which previously had not been recorded in the wild there since 1954. We confirm that *C. stenophylla* is an indigenous species in Guinea, Sierra Leone, and Ivory Coast. *Coffea affinis* was discovered in the wild in Sierra Leone for the first time, having previously been found only in Guinea and Ivory Coast. Prior to our rediscovery, *C. affinis* was last seen in the wild in 1941, although sampling of an unidentified herbarium specimen reveals that it was collected in Guinea-Conakry in 2015. DNA sequencing using plastid and ITS markers was used to: (1) confirm the identity of museum and field collected samples of *C. stenophylla*; (2) identify new accessions of *C. affinis*; (3) refute hybrid status for *C. affinis*; (4) identify accessions confused with *C. affinis*; (5) show that *C. affinis* and *C. stenophylla* are closely related, and possibly a single species; (6) substantiate the hybrid *C. stenophylla* × *C. liberica*; (7) demonstrate the use of plastid and nuclear markers as a simple means of identifying F1 and early-generation interspecific hybrids in *Coffea*; (8) infer that *C. liberica* is not monophyletic; and (9) show that hybridization is possible across all the major groups of key Africa *Coffea* species (Coffee Crop Wild Relative Priority Groups I and II). *Coffea affinis* and *C. stenophylla* may possess useful traits for coffee crop plant development, including taste differentiation, disease resistance, and climate resilience. These attributes would be best accessed via breeding programs, although the species may have niche-market potential via minimal domestication.

## Introduction

Coffee is a globally significant crop that supports a multibillion-dollar global industry (International Coffee Organization (ICO), 2019), over a lengthy value chain from farmer to consumer. Coffee farming alone involves the farming activities of around 100 million people worldwide (Vega et al., 2003). Two species dominate global coffee production: Arabica (*Coffea arabica*) and robusta (*C. canephora*), providing c. 60% and c. 40% of traded coffee, respectively (International Coffee Organization (ICO), 2019). Liberica coffee is cultivated worldwide in small quantities, and is insignificant in terms of global trade, although production in the Philippines and Malaysia can be substantial. Aside from *C. arabica*, *C. canephora*, and *C. liberica*, there are another 121 coffee species known to science (Davis et al., 2006, 2011, 2019). Some of these are used to make the beverage coffee, such as *C. congensis*, *C. eugenioides*, and *C. racemosa*, some have been used in breeding programs, and others have been used as high performing pest and diseases resistant rootstocks (Davis et al., 2019). Many more are used on a small, local scale, or are harvested directly from the wild, in Africa, Madagascar, and Asia. In previous centuries, and particularly at the end of the 1800s and early 1900s, there was considerable interest in, and use of, a range of beverage producing coffee species, more than there is today (Davis et al., 2019). It is also of note that since those times, a substantial proportion of the world’s coffee species diversity has been discovered and named by science, particularly from the 1960s onward (Bridson, 1988; Davis et al., 2006; Davis and Rakotonasolo, 2009). The decline in the interest in these ‘other’ coffee species has been largely due to the overwhelming success of robusta coffee, which was itself transformed from a wild plant, and minor African crop species, to a major global commodity in around 150 years (Davis et al., 2019). Robusta gained market share against Arabica from the early 1900s onward due to its resistance to coffee leaf rust (CLR; *Hemileia vastatrix*) (Wrigley, 1988), a broader agroecological envelope (Davis et al., 2006), higher productivity (Wellman, 1961; Wrigley, 1988), lower purchase price (International Coffee Organization (ICO), 2019), and other specific attributes (Davis et al., 2019). Recently, however, there has been renewed interest in underutilized, forgotten, and little known coffee species, both cultivated and wild, due to their potential to counter specific pests and diseases, and provide resilience in an era of accelerated climate change (Davis et al., 2019). There is also an increasing curiosity in lesser known coffee species from the specialty coffee sector, in its quest to discover new and differentiated sensory experiences in coffee.

Among those of particular interest are two West African species: *C. stenophylla* and *C. affinis*, mainly due to historical reports of a superior taste, particularly for *C. stenophylla* (Cheney, 1925) but also *C. affinis* (De Wildeman, 1904). Given that these two species occur in Upper West Africa at relatively low elevations (see below) there may also be the potential for climate resilience. Both species fall within Coffee Crop Wild Relative Priority Group II, which includes species closely related to the main crop species, for which gene transfer to the crop is proven or assumed (with low to high post-crossing fertility rates) (Davis et al., 2019). Priority Group II includes all African species, apart from the main coffee crop species and their progenitors (*C. arabica*, *C. canephora*, *C. liberica*, and *C. eugenioides*: Priority Group I) and African species of Priority Group III. Priority Group III includes all the short-styled *Coffea* species (previously assigned to the genus *Psilanthus*) from Africa, Asia and Australasia, and all Madagascan species and Mascarene species (Davis et al., 2019).

Our recent knowledge of *C. stenophylla* and *C. affinis* is principally limited to germplasm surveys. *Coffea stenophylla* is recorded as a living plant in several (*ex situ*) coffee research collections (Anthony et al., 2007; Engelmann et al., 2007; Bramel et al., 2017); *C. affinis* is included in the most recent of these reviews (Bramel et al., 2017) but only as an entry based on our knowledge of accepted coffee species (Govaerts et al., 2019). Contemporary evaluations of coffee species diversity (Davis et al., 2006, 2011, 2019; Maurin et al., 2007; Hamon et al., 2017) clearly show that our knowledge of *C. stenophylla* and *C. affinis* is inadequate. Initial review of literature for *C. affinis* showed almost no extra knowledge of this species has been gained since 1937 (Portères, 1937a), with the exception of work in Ivory Coast and Guinea in the 1980s (Berthaud, 1983, 1986; Le Pierrès et al., 1989). It is imperative that we improve our knowledge of these two species, both in cultivation (including any commercial production) and in the wild.

In this study, our main objectives were to elucidate: the current cultivated and wild status of *C. stenophylla* and *C. affinis;* the taxonomic identity and systematic position of the poorly known *C. affinis*; and to assemble available information on crop plant attributes. To achieve these objectives we undertook: (1) a literature review; (2) a survey of herbarium and economic botany collections; (3) field surveys in Sierra Leone, visiting farms, research stations and natural forest locations; and (4) DNA sequencing of recently collected material, historical samples (herbarium and economic botany collection samples), known interspecies hybrids, and their analysis incorporating a reference set of previously published *Coffea* sequences.

## Materials and Methods

### Literature Review

We examined all key literature pertaining to *C. affinis* and *C. stenophylla*. Knowledge of *ex situ* cultivation in research collections was gleaned from published works (Anthony, 1982, 1992; Anthony et al., 2007; Engelmann et al., 2007; Bramel et al., 2017; Davis et al., 2019), supported by personal observations (A. Davis, J. Haggar) and personal communication.

### Review of Herbarium Collections and Economic Botany Collections

Herbarium specimens are well suited to this type of study because they are verifiable in space (location), time (date) and form (species identity), and are often accompanied by additional information on the herbarium label (e.g., ecology, elevation, geology, and uses). We consulted herbarium specimen records from nine herbaria (BM, BR, K, MO, P, UPS, WAG) including those in Sierra Leone (SL, FBC). Herbarium codes follow standard abbreviations (Holmgren et al., 1990; Thiers, 2019). The specimen data was disaggregated into unique records and duplicate specimens. Unique records comprise the combination of collector’s name and number (e.g., *Chillou 2381*) or collector’s name and date (e.g., Cope *s.n.*, 7 iii 1912); *s.n.* is an abbreviation for *sine numero*, and lowercase Roman numerals to represent the month). Duplicate specimens possess the same unique identifier, i.e., they are from the same plant or possibly nearby individuals, but are found on separate herbarium sheets; these may either be found in the same herbarium or across two or more herbaria.

### Fieldwork in Sierra Leone

During 2014 and 2016 we made a request for samples of *C. affinis* and *C. stenophylla* and any atypical coffee morphotypes, from NGOs and farmer associations representing 10,000 coffee farmers across Kenema, Kailahun, and Kono Districts, which represent the major coffee producing region of Sierra Leone. We also visited the Sierra Leone Agricultural Research Institute (SLARI) research collection at Pendembu, Kailahun District (Table 1), to sample putative examples of *C. stenophylla* and *C. affinis*. In addition, 50 A4 posters showing the most obvious morphological differences (leaf shape and size) between the two cultivated coffee species, robusta coffee (*C. canephora*) and Liberica (*C. liberica*) and *C. stenophylla* were printed and distributed to district agriculture offices with coffee farming communities in southern Sierra Leone, between Freetown and Kenema. The aim was to provide an additional means of identifying farms that might be cultivating *C. stenophylla* or *C. affinis*. Visits to sites where *C. stenophylla* had been recorded in cultivation in northern Sierra Leone (based on the herbarium survey) were visited in 2017. In December 2018, we followed up on the poster survey, by visiting five farms that had stated cultivation of *C. stenophylla*. On the same trip, we visited the last known (1954) forest sites for *C. stenophylla* in the Kasewe Hills (Southern Province), and several possible locations: around Freetown (Western Area), near Moyamba Junction (Southern Province), and the forest area of Kambui Hills, adjacent to Kenema (Eastern Province). Follow up visits to the Kambui Hills were made throughout 2019 and in early 2020.

**TABLE 1:** List of material examined, with origin, source of material, and (DNA) identification.

**Accession of *Coffea* as received**	**Origin**	**Material**	**Name on DNA trees, and final identification**
**Sierra Leone cultivated accessions**			
*C.* ?*affinis* [1a] (purple fruits, and purple tinge to stem and leaves)	Sierra Leone. Kangama, Gorama, Kono.	Leaf	*C. canephora* (1) SL cult.
*C.* sp. [2-16 Office]	Sierra Leone, Kenema	Leaf	*C. canephora* (2) SL cult.
*C.* sp. [1-16 Office]	Sierra Leone, Kenema	Leaf	*C. canephora* (3) SL cult.
*C. liberica* (leaves, 30 cm long, and almost as broad) [12a]	Sierra Leone, CEPAH, Kono	Leaf	*C. liberica* (1) SL cult.
*C.* sp. [14-16, Site 1]	Sierra Leone, East Fiama, Kono	Leaf	*C. liberica* (2) SL cult.
*C.* sp. [16-16 Site 3]	Sierra Leone, Fiama, Kono	Leaf	*C. liberica* (3) SL cult.
*C.* sp. [18-16, Site 1]	Sierra Leone, Lei Chiefdom, Kono	Leaf	*C. liberica* (4) SL cult.
*C. arabica* Pendembu, SLARI collection [10a]	Sierra Leone, Pendembu, Kailahun, SLARI collection	Leaf	*C. stenophylla* × *C. liberica* (1) SL cult.
*C. affinis* [8]	Sierra Leone, Pendembu, Kailahun, SLARI collection	Leaf	*C. stenophylla* × *C. liberica* (2) SL cult.
*C. affinis* [9]	Sierra Leone, Pendembu, Kailahun, SLARI collection	Leaf	*C. stenophylla* × *C. liberica* (3) SL cult.
**Sierra Leone wild accessions**			
*C. affinis*	Sierra Leone, Kambui Hills, 2020, *Sarmus.n.* (K)	Seed	*C. affinis* (1) SL
*C. affinis*	Sierra Leone, Kambui Hills, 2020, *Sarmus.n.* (K)	Leaf	*C. affinis* (2) SL
*C. stenophylla*	Sierra Leone, Kasewe Hills	Leaf	*C. stenophylla* (5) SL
*C. stenophylla*	Sierra Leone, Kambui Hills	Leaf	*C. stenophylla* (6) SL
**Kew (K) accessions (museum)**			
*C.* sp. unknown	Guinea-Conakry, Coyah Prefecture, Saliya, 2015, *Couch 757*(K)		*C. affinis* (3) Guinea
*C. liberica*	Uganda, cultivated.	Seed	*C. liberica* (5) Uganda cult.
*C. affinis*	Sierra Leone, Plantation, Sierra Leone, 1902, *Cope* s.n. (K)	Seed	*C.* sp. (1) SL cult.
*C. affinis*	Sierra Leone, Plantation, Sierra Leone, 1902, *Cope* s.n. (K)	Seed	*C.* sp. (2) SL cult.
*C. stenophylla* [1]	Sierra Leone, Botanical Station, 15 Jan 1896	Seed	*C. stenophylla* (1) SL cult.
*C. stenophylla* [2]	Sierra Leone, 1856 (Highland coffee). No. 4	Seed	*C. stenophylla* (2) SL cult.
*C. stenophylla* [4]	Sierra Leone, cult. Brazil	Seed	*C. stenophylla* (7) Brazil cult.
*C. stenophylla* [3]	Sierra Leone, cult. lowland grounds, 1856, No. 3	Seed	No DNA data
**Brazil Accessions**			
*C. arabica* (‘Typica’)	Brazil (cultivated), originally from Ethiopia	Leaf	*C. arabica* (2) cult.
*C. arabica* × *C. racemosa* [Clone 3]	Brazil (cultivated man made hybrid)	Leaf	*C. arabica* × *C. racemosa* (1) Brazil cult.
*C. arabica* × *C. racemosa* [Clone 12]	Brazil (cultivated, artificial hybrid)	Leaf	*C. arabica* × *C. racemosa* (2) Brazil cult.
*C. arabica* × *C. racemosa* × *C. arabica* [IAC1196]	Brazil (cultivated, artificial hybrid)	Leaf	*C. racemosa* × *C. arabica* (backcross) Brazil cult.
*C. racemosa* (putative hybrid parent)	Brazil (cultivated), originally from East Africa	Leaf	*C. racemosa* (1) Brazil cult.
*C. stenophylla*	Brazil (cultivated), originally from Upper West Africa	Lead	*C. stenophylla* (7) Brazil cult.
**Other material**			
*C. liberica*	DR Congo ex Belgian Botanic Gardens (BR) # 19370045 (BR)	Leaf	*C. liberica* (9) DRC

*The taxa in the furthest right-hand column are those used on the phylogenetic trees (Figures 4, 5); to find the accession identifier (as received and as discussed in the text) use left-hand column. For previously sequenced accessions included in Figures 4, 5, see Table 2). Seed, single seed from herbarium sample. Leaf, leaf sample in silica gel.*

### Assembly of DNA Reference Collection

The most taxonomically comprehensive DNA dataset for wild coffee species is for plastid (*trnL*–*F* intron, *trnL*–*F* intergenic spacer (IGS), *rpl16* intron and *accD*–*psaI* IGS) and the internal transcribed spacer (ITS) region of nuclear rDNA (ITS 1/5.8S/ITS 2) (Maurin et al., 2007; Davis et al., 2011). These markers have the ability to distinguish between African coffee species, and identify recently formed hybrids via differential inheritance of plastid and nuclear genomes (Maurin et al., 2007). Thirty-one accessions (Tables 1, 2) were sequenced with the four markers: 14 collected by us in Sierra Leone; nine reference samples from the museum collections of RBG Kew (K), including three for *C. stenophylla*, four for *C. affinis* (including two farmed accessions), and four other coffee species; five samples associated with the production of the artificial hybrid *C. arabica* × *C. racemosa* (Medina Filho et al., 1977a, b); and one unpublished sequence of *C. zanguebariae* (Table 2). The five museum collections of *C. stenophylla* and *C. affinis* were selected to represent authentic material, i.e., that being cultivated at the end of the 19th and beginning of the 20th centuries (respectively) in Sierra Leone. The published reference sequence dataset (Maurin et al., 2007; Davis et al., 2011) included a single verified example of *C. stenophylla*. Two known interspecific hybrids, as identified in a previous study using the same markers (Maurin et al., 2007), were included in the sampling: *C. arabica* (*C. canephora* × *C. eugenioides*) and *C. liberica* × *C. eugenioides* [originally accessioned (Maurin et al., 2007) as *C. heterocalyx*]. Initial analyses were conducted using the study species (see above) and a global data set of African, Madagascar, Mascarene, and Asian coffee species (Maurin et al., 2007; Davis et al., 2011). Following this analysis and confirming general placement of accessions, this was reduced to African taxa, excluding short-styled *Coffea* species (former *Psilanthus*), equating to Coffee Crop Wild Relative (Priority) Groups I and II (Davis et al., 2019).

**TABLE 2:** List of sequence accession data.

***Coffea* species and accession identifier**	**Source**	***ITS***	***trnL–trnF***	***rpl16***	***accD–psaI***
*C. affinis* (1) SL	Sierra Leone, Kambui Hills	**MT250043**	**MT274308**	**MT274311**	–
*C. affinis* (2) SL	Sierra Leone, Kambui Hills	**MT250044**	**MT274309**	**MT274312**	**MT274306**
*C. affinis* (3) Guinea	Guinea-Conakry, Saliya, 2015, *Couch 757* (K)	**MT250045**	**MT274310**	**MT274313**	**MT274307**
*C.* sp. (1) SL cult.	Sierra Leone (cult.) *	**MN719945**	**MN715153**	**MN715208**	**MN715181**
*C.* sp. (2) SL cult.	Sierra Leone (cult.) *	**MN719946**	**MN715154**	**MN715209**	**MN715182**
*C. anthonyi*	DR Congo	DQ153620	DQ153856	DQ153738	DQ153489
*C. arabica* (1)	Mauritius (cult.)	DQ153609	DQ153845	DQ153727	DQ153478.1
*C. arabica* (2) cult.	Sierra Leone (cult.)	**MN719947**	**MN715155**	**MN715210**	**MN715183**
*C. arabica* × *C. racemosa* (1) Brazil cult.	Brazil (cult.)	**MN719948**	**MN715156**	**MN715211**	**MN715184**
*C. arabica* × *C. racemosa* (2) Brazil cult.	Brazil (cult.)	**MN719949**	**MN715157**	**MN715212**	**MN715185**
*C. arabica* × *C. racemosa* (backcross) Brazil cult.	Brazil (cult.)	**MN719962**	**MN715170**	**MN715225**	**MN715198**
*C. bakossi*	Cameroon	DQ153599	DQ153835	DQ153717	DQ153468
*C. brevipes*	Cameroon	DQ153591	DQ153827	DQ153709	DQ153460
*C. bridsoniae*	Tanzania	DQ153584	DQ153822	DQ153704	DQ153455
*C. canephora* (1) SL cult.	Sierra Leone (cult.)	**MN719950**	**MN715158**	**MN715213**	**MN715186**
*C. canephora* (2) SL cult.	Sierra Leone (cult.)	**MN719951**	**MN715159**	**MN715214**	**MN715187**
*C. canephora* (3) SL cult.	Sierra Leone (cult.)	**MN719952**	**MN715160**	**MN715215**	**MN715188**
*C. canephora* (4) SL cult.	Cameroon (cult.)	DQ153593	DQ153829	DQ153711	DQ153462
*C. congensis*	Cameroon	DQ153632	DQ153834	DQ153716	DQ153467
*C. costatifructa*	Tanzania	DQ153604	DQ153840	DQ15372	DQ153473
*C. eugenioides*	Tanzania	DQ153588	DQ153824	DQ153706	DQ153457
*C. fadenii*	Tanzania	DQ153574	DQ153813	DQ153695	DQ153446
*C. heterocalyx*	Cameroon	DQ153594	DQ153830	DQ153712	DQ153463
*C. humilis*	Ivory Coast	DQ153611	DQ153847	DQ153729	DQ153480
*C. kapakata*	Angola	DQ153596	DQ153832	DQ153714	DQ153465
*C. kihansiensis*	Tanzania	DQ153583	DQ153821	DQ153703	DQ153454
*C. kimbozensis*	Tanzania	DQ153575	DQ153814	DQ153696	DQ153447
*C. kivuensis*	Tanzania	DQ153612	DQ153848	DQ153730	DQ153481
*C. liberica* (1) SL cult.	Sierra Leone (cult.)	**MN719956**	**MN715164**	**MN715219**	**MN715192**
*C. liberica* (2) SL cult.	Sierra Leone (cult.)	**MN719957**	**MN715165**	**MN715220**	**MN715193**
*C. liberica* (3) SL cult.	Sierra Leone (cult.)	**MN719958**	**MN715166**	**MN715221**	**MN715194**
*C. liberica* (4) SL cult.	Sierra Leone (cult.)	**MN719959**	**MN715167**	**MN715222**	**MN715195**
*C. liberica* (5) Uganda cult.	Uganda	**MN719960**	**MN715168**	**MN715223**	**MN715196**
*C. liberica* (6) CAR	Central African Rep.	DQ153603	DQ153839	DQ153721	DQ153472
*C. liberica* (7) DRC	DR Congo	**MN719953**	**MN715161**	**MN715216**	**MN715189**
*C. liberica* (8) DRC	DR Congo	**MN719954**	**MN715162**	**MN715217**	**MN715190**
*C. liberica* (9) DRC	DR Congo	**MN719955**	**MN715163**	**MN715218**	**MN715191**
*C. liberica* × *C. eugenioides*^+^	DR Congo	DQ153623	DQ153859	DQ153741	DQ153492
*C. lulandoensis*	Tanzania	DQ153580	DQ153819	DQ153701	DQ153452
*C. magnistipula*	Cameroon	DQ153640	DQ153876	DQ153758	DQ153509
*C. mayombensis*	Cameroon	DQ153592	DQ153828	DQ153710	DQ153461
*C. mongensis*	Tanzania	DQ153576	DQ153815	DQ153697	DQ153448
*C. montekupensis*	Cameroon	DQ153590	DQ153826	DQ153708	DQ153459
*C. mufindiensis*	Tanzania	DQ153577	DQ153816	DQ153698	DQ153449
*C. pocsii*	Tanzania	DQ153582	DQ153820	DQ153702	DQ153453
*C. pseudozanguebariae*	Tanzania	DQ153578	DQ153817	DQ153699	DQ153450
*C. racemosa* (1) Brazil cult.	Brazil (cult.)	**MN719961**	**MN715169**	**MN715224**	**MN715197**
*C. racemosa* (2) Moz	Mozambique	DQ153627	DQ153863	DQ153745	DQ153496
*C. racemosa* (3) Moz	Mozambique	DQ153595	DQ153831	DQ153713	DQ153464
*C. racemosa* (4) Moz	Mozambique	DQ153628	DQ153864	DQ153746	DQ153497
*C. rhamnifolia*	Somalia*	DQ153589	DQ153825	DQ153707	DQ153458
*C. salvatrix*	Mozambique	DQ153622	DQ153858	DQ153740	DQ153491
*C. schliebenii*	Tanzania	DQ153587	DQ153823	DQ153705	DQ153456
*C. sessiliflora*	Tanzania	DQ153579	DQ153818	DQ153700	DQ153451
*C. stenophylla* (1) SL cult.	Sierra Leone (cult.)*	**MN719966**	**MN715174**	**MN715228**	**MN715201**
*C. stenophylla* (2) SL cult.	Sierra Leone (cult.)*	**MN719967**	**MN715175**	**–**	**MN715202**
*C. stenophylla* (3) IC	Ivory Coast*	DQ153597	DQ153833	DQ153715	DQ153466
*C. stenophylla* (4) IC	Ivory Coast	**MN719968**	**MN715176**	**MN715229**	**MN715203**
*C. stenophylla* (5) SL	Sierra Leone	**MN719964**	**MN715172**	**MN715227**	**MN715200**
*C. stenophylla* (6) SL	Sierra Leone	**MN719965**	**MN715173**	**–**	**–**
*C. stenophylla* (7) Brazil cult.	Brazil (cult.)	**MN719969**	**MN715177**	**MN715230**	**MN715204**
*C. stenophylla* × *C. liberica* (1) cult.	Sierra Leone (cult.)	**MN719972**	**MN715180**	**MN715233**	**MN715207**
*C. stenophylla* × *C. liberica* (2) cult.	Sierra Leone (cult.)	**MN719970**	**MN715178**	**MN715231**	**MN715205**
*C. stenophylla* × *C. liberica* (3) cult.	Sierra Leone (cult.)	**MN719971**	**MN715179**	**MN715232**	**MN715206**
*C. togoensis*	Togo*	DQ153607	DQ153843	DQ153725	DQ153476
*C. zanguebariae*	Mozambique	**MN719963**	**MN715171**	**MN715226**	**MN715199**

Newly sequenced material in bold. See Table 1 for details of accessions sequenced for this study. See Maurin et al. (2007) for details of previously sequenced material. * Accessions sequenced from herbarium material; ^+^Accession listed as *Coffea heterocalyx* in Maurin et al. (2007).

### DNA Extraction, Sequencing, and Data Analysis

Total DNA was extracted from silica dried leaves, fresh seeds, and seeds from herbarium specimens and other archival material (Tables 1, 2) using a modified CTAB method (Doyle and Doyle, 1987) and purified using the QIAquick PCR purification kit (QIAGEN). Genetic variation among the accessions was assessed by employing four regions: nuclear internal transcribed spacers (ITS1 and ITS2), plastid *trnL*–*trnF* (*trnL* intron and *trnL*–*trnF* intergenic spacer), *rpl16* intron and *accD*–*psaI* intergenic spacer. Amplifications were carried out following the protocol of Maurin et al. (2007); PCR products were purified using QIAquick PCR purification kit (QIAGEN) and sequenced following the methods employed by Maurin et al. (2007). Capillary electrophoresis was conducted on an ABI3730 DNA Analyzer (Applied Biosystems). Sequencing results were inspected in GENEIOUS v. 8.1.7 (Kearse et al., 2012). Newly sequenced accessions and unpublished sequences held at RBG Kew were referenced against GenBank accessions of *Coffea* species (Maurin et al., 2007; Davis et al., 2011). The sequences were aligned using MUSCLE (Edgar, 2004), as implemented in GENEIOUS. Gaps were treated as missing data and ambiguities were scored with IUPAC ambiguity codes. The model of character evolution was assessed in jModelTest v. 2.1.10 (Posada, 2008). Relationships among the taxa were reconstructed in MrBayes v. 3.2.7a (Huelsenbeck and Ronquist, 2001; Ronquist and Huelsenbeck, 2003) as implemented on the CIPRES Scientific Gateway v3.3; *C. rhamnifolia* was used as the outgroup. Analyses were conducted separately for the ITS and the plastid DNA datasets and regions difficult to align were excluded from the phylogenetic analysis. We also conducted a separate analysis on the concatenated ITS/plastid matrix for the Upper Guinea (UG) clade (including *C. togoensis*, *C. affinis*, and C. *stenophylla*), with *C. canephora* and *C. liberica* as outgroups. MCMC sampling was performed with two runs and four chains for 2 × 10^7^ generations, with a sampling frequency of 1,000 and a relative burn-in of 25%; specified model of character evolution was GTR+G. Convergence was visually assessed with Tracer v. 1.6 (Rambaut et al., 2013), by combining trace files to confirm mixing and high effective sampling size (EES). Maximum clade credibility trees were drawn in FigTree v. 1.4.4 (Rambaut, 2018). Clade and species alliance terminology follow Maurin et al. (2007) and Davis et al. (2011).

## Results

### Literature Review

#### *Coffea stenophylla* (Highland Coffee of Sierra Leone, Rio-Nunez Coffee, Senegal Coffee, Sierra Leone Coffee) (Figures 1, 2A)

**FIGURE 1:**
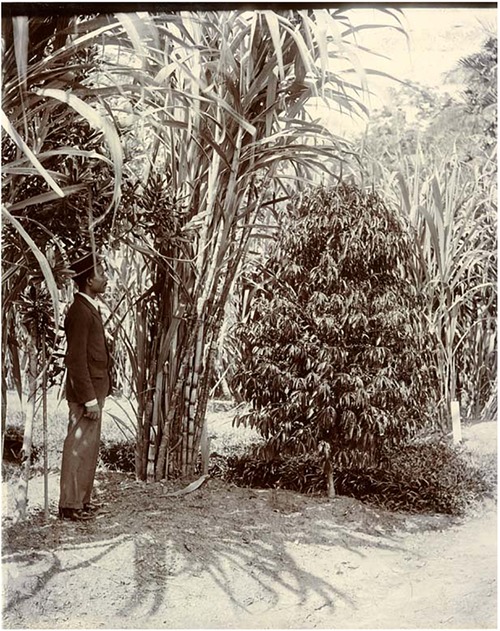
*Coffea stenophylla*, cultivated in Trinidad Botanical Garden, with Demerara sugarcanes, photograph taken around 1900. The man in the photograph is 5 ft. 8 in. (1.72 m) tall. Image: Royal Botanic Gardens, Kew.

Knowledge of *C. stenophylla* and its commercial potential dates back to at least 1794, based on reports by Adam Afzelius (1750–1837), who worked in, and collected plants from, Sierra Leone (Hiern, 1876). *Coffea stenophylla* was described as new to science in 1834 (Don, 1834) and was characterized on the basis of having narrow leaves (hence the species epithet) and black fruits (most coffee species have red fruits). Don (1834) stated that is was a ‘Native of Sierra Leone, where it is cultivated’… and that ‘The seeds of this species are roasted and used as the common coffee, and are even considered superior to it.’ De Wildeman (1904) reported *C. stenophylla* as indigenous in Guinea (in the forests that border the Southern Rivers area) and Ivory Coast. From at least the 1850s, the seeds of *C. stenophylla* were disseminated from Sierra Leone, with the accompanying vernacular names of ‘Highland coffee of Sierra Leone’ or ‘Sierra Leone coffee’ (Hooker, 1896). Commercial (cultivated) samples reached the Royal Botanic Gardens, Kew (England) in 1856 (specimens in the Economic Botany Collection, Kew). According to Chevalier (1929), *C. stenophylla* was being cultivated in quantity in Sierra Leone in the 1890s (c. 1893), and in Guinea, to the extent that is was exported as a commercial product to France. In France it apparently received an exceptionally favorable market price (Scott Elliot, 1893). Living material (seeds) of *C. stenophylla* were sent to the Royal Botanic Gardens, Kew in May 1894, and from here it was sent to India, Sri Lanka (then known as Ceylon), Trinidad (Figure 1), and Java (Cheney, 1925; Scott Elliot, 1893). From Guinea is was sent to Vietnam (Chevalier, 1929) and probably other countries under French colonial rule at that time. In 1904, De Wildeman (1904) provided a summary of the cultivation of *C. stenophylla* in Guinea, where it seems to have been cultivated in some quantity as Rio-Nunez coffee, after the Nunez River (a major river in Guinea). It was also cultivated in Ghana, Senegal (where it was known as Senegal coffee), and Ivory Coast, possibly through early intervention by the Portuguese (Haarer, 1962), and in Uganda (Tothill, 1940). Chevalier (1929), states that the export of *C. stenophylla* in Sierra Leone and Guinea amounted to around three to five tons (3,000 to 5,000 kg) per year, although this does not include the amount of coffee consumed in these producing countries, which may have been substantial. *Coffea stenophylla* appears to have been a prominent feature of agriculture in Sierra Leone up until at least the 1920s (Dudgeon, 1922), but it may have been in decline after that time (Chevalier, 1929), perhaps due to a fall in coffee prices (Dudgeon, 1922). Elsewhere, despite all the reports of an excellent flavor (Don, 1834; Scott Elliot, 1893; De Wildeman, 1904, 1906b; Watt, 1908; Macmillan, 1914; Dudgeon, 1922; Cheney, 1925; Chevalier, 1929; Wellman, 1961; Haarer, 1962) and a range of potential agronomic attributes (see below) it did not prevail as a coffee crop species. Chevalier (1929) reported that although it was considered by many to be an exquisite coffee (“Suivant beaucoup de dégustateurs, c’est un café exquis”) it was not widespread in global cultivation, due to low yields. Likewise, despite being introduced to Uganda in 1919 and then again in 1931, small bean size and low yield prevented it from being a sustainable coffee crop plant (Tothill, 1940). Despite this, a good agronomic performance at low elevations (e.g., c. 150 m) has been reported for *C. stenophylla* (Watt, 1908; Cheney, 1925; Bramel et al., 2017), and there is a report of potential resistance to CLR (Cheney, 1925). Given that the natural, and once-cultivated, environments of these two species in Upper West Africa were at relatively low elevations (150–610 m) (Watt, 1908) and that *C. stenophylla* is reported to withstand dry conditions (Wellman, 1961; Wrigley, 1988), there may also be some resilience to high temperatures and low rainfall, compared to the main crop species.

**FIGURE 2:**
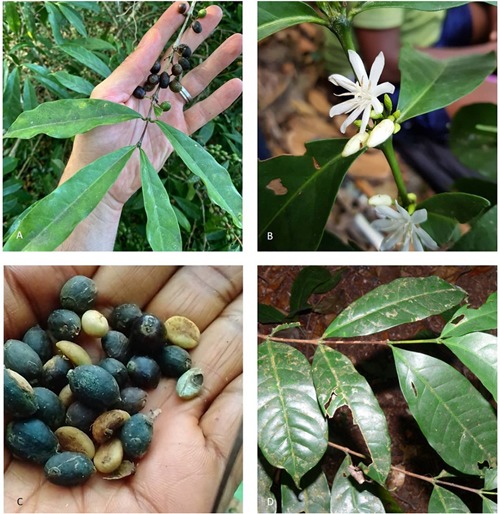
*Coffea affinis* and *C. stenophylla*. **(A)** *C. stenophylla* in fruit, at Centre National de Recherche Agronomique (CNRA), Ivory Coast (image: Charles Denison); **(B)** *C. affinis* in flower; **(C)** *C. affinis*, fruits and seeds (partially dried); **(D)** *C. affinis*, leaves. Images (**B–D)**, from Kambui Hills, Sierra Leone (images: Daniel Sarmu).

Since the 1940s, most of the literature on *C. stenophylla* has been restricted to the recycling of information from previous publications (Wellman, 1961; Haarer, 1962; Wrigley, 1988; Stoffelen, 1998; Davis et al., 2006). The exceptions to this are the reports of dedicated coffee collection missions in Upper West Africa undertaken in the 1980s. Reporting on various missions to Ivory Coast between 1984 and 1987, Le Pierrès et al. (1989) record two wild populations of *C. stenophylla* from the main forest block of the Sud-Est region of Ivory Coast (north east of Abidjan); and from Guinea, 114 examples of small scale cultivation of this species in the gardens of local houses (between Boffa and Boke, and around Boke). Berthaud (1986) demonstrated the existence of populations of *C. stenophylla* in Ivory Coast, from Ira Forest (Forêt L’Ira), three other localities (populations) in the east of the country. In addition, Berthaud (1983) recorded this species at a dry forest site in the Ouellé area in western Ivory Coast. Berthaud (1986) reported *C. stenophylla*, *C. liberica*, and *C. canephora* in Ira Forest (Forêt L’Ira); *C. stenophylla* was restricted to the upper, drier parts of the hills, whereas the other two species were found in the valley bottoms (lower, wetter areas).

#### *Coffea affinis* (Kamaya Coffee)

This species was first reported in c. 1900 from the coffee research garden of M. Boery in Guinea, having been originally collected from nearby native forests (De Wildeman, 1904). On first inspection, De Wildeman considered these plants to be similar in many characteristics to *C. stenophylla* (i.e., the presence of black fruits, rather than the usual red) but different in other respects and particularly leaf size and shape (De Wildeman, 1904). When De Wildeman visited Guinea (De Wildeman, 1904) to observe these plants, which were being grown collectively as Rio-Nunez coffee, he declared that there were two species, *C. stenophylla* and another species, which he named as a new to science: *C. affinis*. According to De Wildeman (1904) *C. affinis* was akin to *C. stenophylla* in the color and shape of the fruit, and shape of the seeds, but differed in its vegetative characters (e.g., stems, leaves, stipules) and mainly by its larger leaves. De Wildeman (1904) believed that *C. affinis* was of considerable importance as new coffee crop species, due to its vigor, the quality (high value) of the coffee, and general resistance to disease (compared to Arabica coffee). A contemporaneous photograph of *C. affinis* (De Wildeman, 1906b) shows a coffee plant that differs from the narrow-leaved *C. stenophylla* by having larger, broader leaves.

**FIGURE 3:**
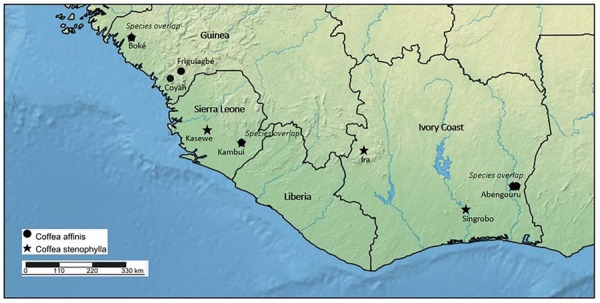
Distribution map of *Coffea affinis* and *C. stenophylla* based on herbarium and literature survey, and fieldwork. Labels beneath species symbols indicate general collection sites [Boké, Friguiagbé, Coyah (Saliya Forest Reserve), Kasewe (Kasewe Hills Forest Reserve, near Moyamba), Kambui (Kambui Hills Forest Reserve, near Kenema, Ira [Ira Forest (Forêt L’Ira), north of Man], Abengouru and Singrobo)]. *Species overlap*, indicates where *C. affinis* and *C. stenophylla* occur in the same location.

Contrary to the viewpoints of De Wildeman (1904, 1906b), Chevalier (1905) suggested that *C. affinis* was native in Sierra Leone, as he had no knowledge of it growing wild in Guinea. Subsequently, Chevalier (1929) considered *C. affinis* to be a hybrid between *C. liberica* and *C. stenophylla*. The potential hybrid status of *C. affinis* was discussed at length by Portères (1937a), who argued against a hybrid origin, particularly in relation to a new coffee plant he considered indigenous to the Ivory Coast, known locally as ‘Kamaya.’ He drew a close association between ‘Kamaya’ and *C. affinis*, but owing to the uncertainty over the application of *C. affinis*, decided to name this plant *C. stenophylla* var. *camaya*. Portères (Portères, 1937a) reported that *C. stenophylla* var. *camaya* was found as single example in a coffee plantation near Abengourou (Figure 3), but that it originated from the wild at a nearby location close to Niabli (6° 39′ N 3° 16′ W) and that a few small plantations were established in Ivory Coast. A decade later, Chevalier (1947) suggested that *C. stenophylla* var. *camaya* and *C. affinis* were the same species, and in contrast to his earlier report was only found in its wild state in Ivory Coast. Wellman (1961) considered *C. affinis* to be indigenous to Guinea and Ivory Coast, and suggested that it was a fixed mutation of *C. stenophylla*. Other workers (Cramer, 1957; Stoffelen, 1998) referred back to the earlier opinion of Chevalier (1905), i.e., that *C. affinis* is a hybrid between *C. liberica* and *C. stenophylla* (Davis et al., 2006).

### Herbarium Collection Survey

For *C. affinis* the herbarium survey yielded 12 unique records (seven cultivated and five wild records) with a total of 28 herbarium specimens (including duplicates), and for *C. stenophylla* 50 unique records (29 cultivated, 12 wild, and 9 with no data) and 72 herbarium specimens. Herbarium records associated with these species include the hybrid *C. liberica* × *C. stenophylla*, of which there were two unique records, both from cultivated material found in research collections. Compared to many other coffee species the number of herbarium specimens is low, especially for *C. affinis*. We examined two commercial seed collections of *C. stenophylla*, one with and one without parchment (pre-milling stage, with endocarp attached) and one of clean (green, pre-roasted) coffee (endocarp removed), and one fruit collection (whole, sun-dried fruits) from the Economic Botany Collection of the Royal Botanic Gardens, Kew (K). Examination of herbarium specimens confirms many aspects of the literature survey (see above).

From the herbarium survey, *C. stenophylla* is confirmed as an indigenous (wild) species of Guinea, Sierra Leone and Ivory Coast (Davis et al., 2006), and that it was also farmed and otherwise cultivated (e.g., research stations and farms) in these countries, as per the literature (see above). Most of the herbarium specimens date from the late 1800s and early 1900s. The most recent collections for these countries are as follows: Guinea (from the wild, 1941; from (small-scale) farm cultivation 1961); Sierra Leone [wild, 1954; from (small-scale) farm cultivation 1963]; Ivory Coast (wild 1932; from farms, no data). By comparison, the literature survey reveals small scale cultivation of *C. stenophylla* in Guinea and Ivory Coast, in the mid to late 1980s (Berthaud, 1983, 1986; Le Pierrès et al., 1989). Herbarium data also show that this species was cultivated in coffee research collections, and other germplasm collections, in Africa (Ivory Coast, Guinea, Nigeria, Sao Tomé, Sierra Leone, Tanzania, Ghana, and the Democratic Republic of Congo) and in Asia (Vietnam, Java).

The herbarium survey reveals that *C. affinis* is as an indigenous (wild) species of Guinea and Ivory Coast (De Wildeman, 1904). Chevalier could not find any evidence of its wild status in Guinea, referring only to cultivated material from the gardens of Conakry and Cameyenne (Chevalier, 1905), but herbarium data provide clear evidence of collections from natural forests in Guinea (see below). In contrast to the views of Chevalier (1905), and even more recent opinion (Davis et al., 2006) we could not find any evidence of wild *C. affinis* in Sierra Leone, which is also the case for the literature survey (but see Fieldwork in Sierra Leone, below). Herbarium specimens exist that were collected from a coffee plantation [Cope *s.n.*, 7 iii 1912 (K)], labeled as *C. affinis*, which has the key leaf characteristics of this species but the flowers are absent, and the fruit color is not noted. Regarding the native status of *C. affinis* in Guinea, there are two collections in the Paris herbarium (P) collected from the environs of Boké (Boké Prefecture) that are identifiable as *C. affinis* and the labels clearly state that the plants were spontaneous (wild): Chillou *s.n*. 20 xii 1923 (three duplicates); Chillou *s.n.*, 17 xii 1923 (two duplicates); a third specimen collected from Friguiagbé (Kindia Prefecture), by the same collector (Chillou *2381*, 3 ii 1941) is also likely to be spontaneous, although the native/cultivated status is not indicated on the specimen. The most recent collections for these countries are as follows: Guinea (from the wild, 1941; from farms, 1905); Sierra Leone (from a coffee plantation, 1912); Ivory Coast (wild 1930; from farms 1934).

### Fieldwork in Sierra Leone

A collection of 20 samples (leaf samples, images, and DNA samples) were made between 2014 and 2016 resulting from our request for samples of *C. affinis* and *C. stenophylla* and of any atypical coffee morphotypes (see the section “Materials and Methods”). Of these, seven were selected for DNA analysis (Table 1); the remaining samples conformed to regular variants of *C. robusta* and *C. liberica*. Three samples of putative *C. stenophylla* and *C. affinis* coffee were collected from the SLARI research collection (Table 1). The 10 samples were either considered as potential candidates for *C. stenophylla* or *C. affinis*, or hybrids between these species. Visits to sites (in 2017) where *C. stenophylla* had been recorded in cultivation in northern Sierra Leone failed to produce any coffee sightings. In December 2018, we followed up on the farm survey, visiting five farms that had stated cultivation of *C. stenophylla*, but no plants of this species or *C. affinis* were located (only *C. canephora*). Our visit to Kasewe Hills (Southern Province) resulted in the collection of a single sterile (no flowers or fruits) immature plant, which we preliminary identified as *C. stenophylla*. We did not find any plants matching *C. stenophylla* in forest locations within the Western Peninsula National Park (near Freetown) or near Moyamba Junction, but located a small population (with mature trees up to 7 m tall) matching this species in the forested area of Kambui Hills. At both localities the plants were collected in humid evergreen (lowland) forest at c. 400 m elevation — on a ridge top in the case of Kasewe Hills and on the side of a ridge on steeply sloping ground at Kambui Hills. Further visits to Kambui Hills throughout 2019 and early 2020 yielded further *C. stenophylla*, and trees provisionally identified as *C. affinis*, in both flower and fruit (Figures 2B–D). DNA samples from the two locations (Kasewe Hills and Kambui Hills) were added to the DNA analyses (see below and Table 1).

### DNA Analyses

A total of 120 sequences from four DNA regions, from 31 accessions, were generated for this study and their sequences deposited in GenBank (with NCBI accession numbers; see Table 2); 35 species-level reference sequences were downloaded from GenBank, from two previous studies (Maurin et al., 2007; Davis et al., 2011). The ITS alignment had a length of 804 bp, whereas the concatenated plastid dataset (*trnL*–*trnF*, *rpl16* and *accD*–*psaI*) had a total length of 3253 bp. A merged ITS/plastid matrix for a subset of species containing members of the Upper Guinea (UG) Clade, plus two outgroup species, had a length of 3989 bp. Some sequences were missing due to poor sequencing quality: *rpl16* in *C. stenophylla* (2) SL cult. and *C. stenophylla* (6) SL; and *accD*–*psaI* in *C. stenophylla* (2) SL cult. and *C. affinis* (1) SL. Accession information is given in Tables 1, 2.

#### ITS Analysis (Figure 4)

**FIGURE 4:**
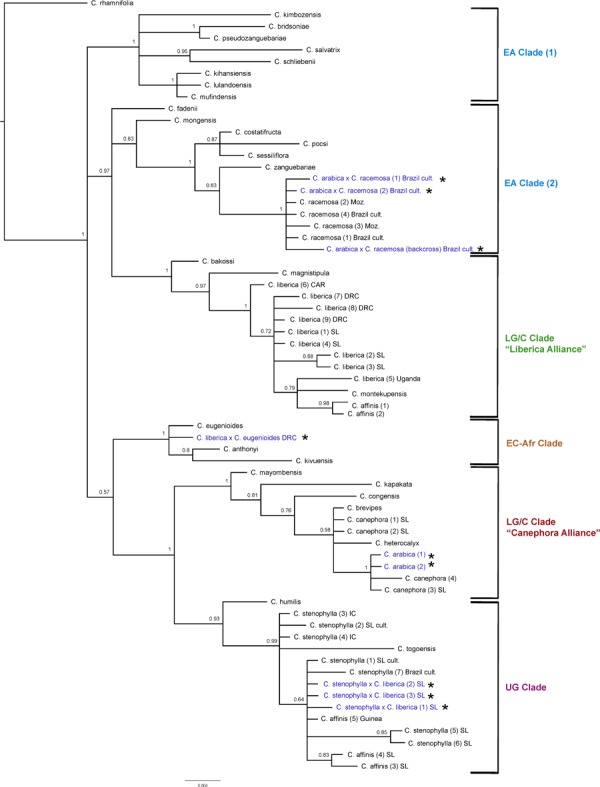
ITS maximum clade credibility tree. Bayesian posterior probabilities are indicated above branches. See Tables 1, 2 for accession information. Country abbreviations: CAR, Central African Republic; DRC, Democratic Republic of Congo; IC, Ivory Coast; Moz, Mozambique; SL, Sierra Leone. Clade terminology follows Maurin et al. (2007) and Davis et al. (2011): EA, East Africa; LG/C, Lower Guinea/Congolian; EC-Afr, East-Central Africa; UG, Upper Guinea. All known and identified interspecies hybrids are marked in blue text and with a star (*).

The results obtained are consistent with previously obtained ITS analysis (Maurin et al., 2007; Davis et al., 2011), in terms of species relationships and their placement into geographically delimited clades. All tetraploid hybrids (4*n* = 44) between *C. arabica* and *C. racemosa* are placed in the East African (EA) Clade (2) with *C. racemosa* (BS = 1). The diploid hybrid (2*n* = 22) *C. liberica* × *C. eugenioides* is placed (BS = 1) with species of the East-Central Africa (EC-Afr) Clade, in an unresolved position with *C. eugenioides*. The natural tetraploid hybrid (4*n* = 44) *C. arabica* (two accessions) is placed with species of the Lower Guinea/Congolian (LG/C) Clade in the ‘Canephora Alliance’ (BS = 1), sister to two accessions of *C. canephora* (BS = 1). Specimens of *C. stenophylla* collected from the wild in Sierra Leone [specimens (5) SL & (6) SL], other wild accessions of this species, and wild accessions of *C. affinis* from Sierra Leone, all fall within the Upper Guinea (UG) Clade (BS = 0.93) with *C. humilis* and *C. togoensis* [specimens (1) SL & (2) SL]. Three cultivated collections from Sierra Leone originally accessioned as *C. arabica* and *C. affinis* (×2) were placed in the Upper Guinea (UG) Clade (labeled in Figure 4 as *C. liberica* × *C. stenophylla* SL cult. (1), (2), and (3). The historical accessions of farmed *C. affinis* from Sierra Leone (*C. affinis* in Table 1) were not placed with *C. stenophylla* or *C. affinis*, but in a clade with *C. liberica* and *C. montekupensis* (BS = 0.79), within the LG/C Clade [labeled as *C*. sp. (1) and *C*. sp. (2) in Figure 4].

#### Plastid Analysis (Figure 5)

**FIGURE 5:**
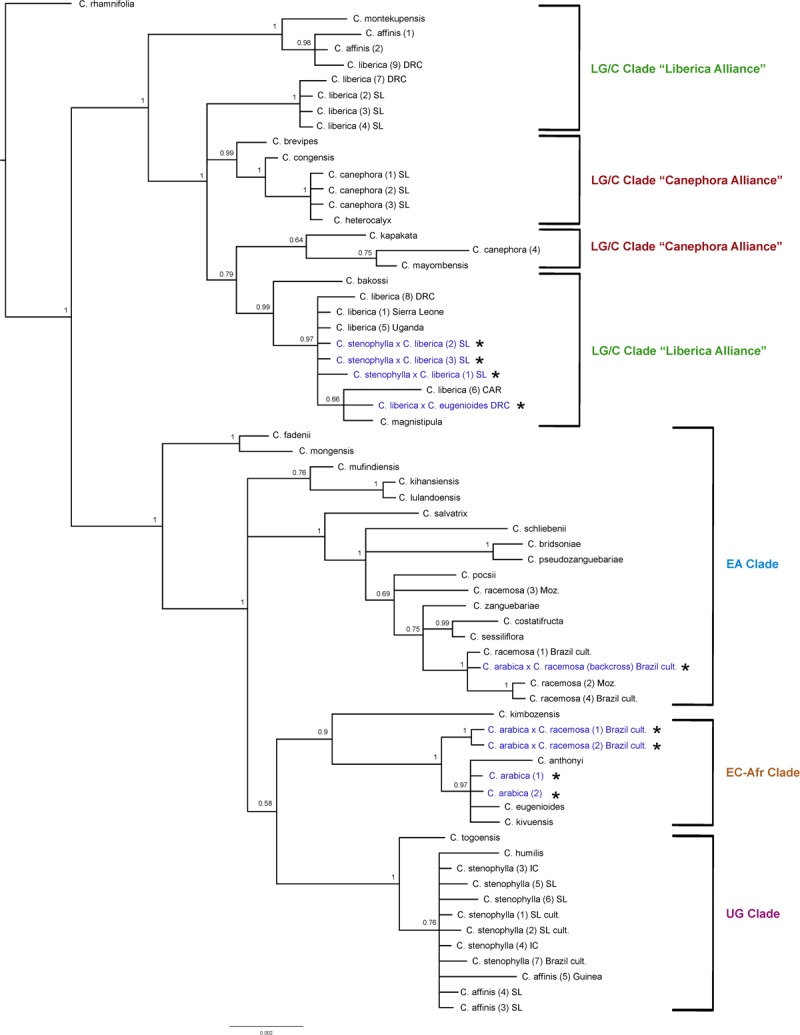
Plastid maximum clade credibility tree. Bayesian posterior probabilities are indicated above branches. See Tables 1, 2 for accession information. Country abbreviations: CAR, Central African Republic; DRC, Democratic Republic of Congo; IC, Ivory Coast; Moz, Mozambique; SL, Sierra Leone. Clade terminology follows Maurin et al. (2007) and Davis et al. (2011): EA, East Africa; LG/C, Lower Guinea/Congolian; EC-Afr, East-Central Africa; UG, Upper Guinea. All known and identified interspecies hybrids are marked in blue text and with a star (*).

The results obtained are consistent with previously obtained plastid analysis using the same markers (Maurin et al., 2007; Davis et al., 2011), in terms of the relationships between species and their placement into geographically delimited clades. The F1 tetraploid hybrids (4*n* = 44) *C. arabica* × *C. racemosa* are placed in the East-Central Africa (EC-Afr) Clade, sister to a clade comprising *C. arabica*, *C. eugenioides*, *C. kivuensis*, and *C. anthonyi*. In contrast to the ITS analysis, the backcrossed *C. arabica* × *C. racemosa* is placed in the EA clade with wild and cultivated *C. racemosa* accessions. *Coffea liberica* × *C. eugenioides* is placed in one of the Lower Guinea/Congolian (LG/C) Clades with two species, *C. liberica* and *C. magnistipula* (BS = 0.66) in an unresolved position with three *C. liberica* accessions (BS = 0.99). The tetraploid (4*n* = 44) hybrid species *C. arabica* is placed with species of the EC-Afr Clade, viz. *C. eugenioides*, *C. kivuensis*, and *C. anthonyi* (BS = 0.97). Three cultivated collections from Sierra Leone originally accessioned as *C. arabica* and *C. affinis* (×2) were placed within one of the two LG/C clades [labeled in Figure 5 as *C. liberica* × *C. stenophylla* SL cult. (1), (2), and (3)] in an unresolved position in a clade with various *C. liberica* accessions (BS = 0.97), and a subclade of *C. liberica* × *C. eugenioides, C. liberica* and *C. magnistipula* (BS = 0.66).

All wild and cultivated accessions of *C. stenophylla*, and wild accessions of *C. affinis* fall within the UG Clade (BS = 1), which includes *C. togoensis* and *C. humilis*. A separate clade containing only all wild and cultivated accessions of *C. stenophylla* and all wild species of *C. affinis*, but including *C. humilis* fall within and in an unresolved clade (BS = 0.76). Our historical accessions of farmed *C. affinis* (*C. affinis* in Table 1) from Sierra Leone are not placed with *C. stenophylla* or *C. affinis*, but in a clade with *C. liberica* (BS = 0.98), sister to *C. montekupensis* (BS = 1), within one of the two LG/C clades [accessions are labeled as *C*. sp. (1) and *C*. sp. (2) in Figure 5].

#### Incongruence Between ITS and Plastid Trees

There are several points of substantial incongruence between the ITS and plastid analyses (Figures 4, 5), including those taxa of known (manmade) or proven (via DNA study) hybrid origin: *C. arabica* (*C. arabica* × *C. eugenioides* (Maurin et al., 2007), *C. arabica* × *C. racemosa* (Medina Filho et al., 1977a, b), and *C. liberica* × *C. eugenioides* (Maurin et al., 2007). These incongruencies are anticipated based on the knowledge, DNA and otherwise, that they are hybrids. In this study we identified three samples, originally accessioned (Table 1) as *C. arabica* and *C. affinis* (× 2), that were substantially incongruent in each analysis. Given the specific positions of these accessions in the analyses (see ITS and plastid results, and Figures 4, 5), and their morphological features, we suggest that they represent the hybrid *C. stenophylla* × *C. liberica*. All known and identified interspecies hybrids are marked in the phylogenetic trees (Figures 4, 5) in blue text and with a star (^∗^).

#### Combined Analysis for the Upper Guinea (UG) Clade (Figure 6)

Combining ITS and plastid data sets for all members of the Upper Guinea (UG) clade, *C. humilis*, *C. togoensis*, *C. affinis*, and *C. stenophylla*, produced relationships congruent with previous analyses using the same data (Maurin et al., 2007; Davis et al., 2011). The four species of the UG Clade are monophyletic (BS = 1), with *C. humilis* sister to *C. togoensis, C. affinis* and *C. stenophylla* (BS = 1); *C. affinis* and *C. stenophylla* form a clade (BS = 0.88) but each species is not monophyletic. The Kambui Hills accessions of *C. affinis* [(1) SL and (2) SL] and *C. stenophylla* accessions from Kasewe Hills and Kambui Hills [(5) SL and (6) SL, respectively] are monophyletic (BS = 0.92), and are sister to two accessions of *C. stenophylla* [(3) IC and (4) IC] from Ivory Coast (BS = 0.76). The *C. affinis* accession [(3) Guinea] from Guinea falls (BS = 0.78) with *C. stenophylla* accessions [(1) SL cult. and (7) Brazil cult], which originate from unknown localities in Sierra Leone.

**FIGURE 6:**
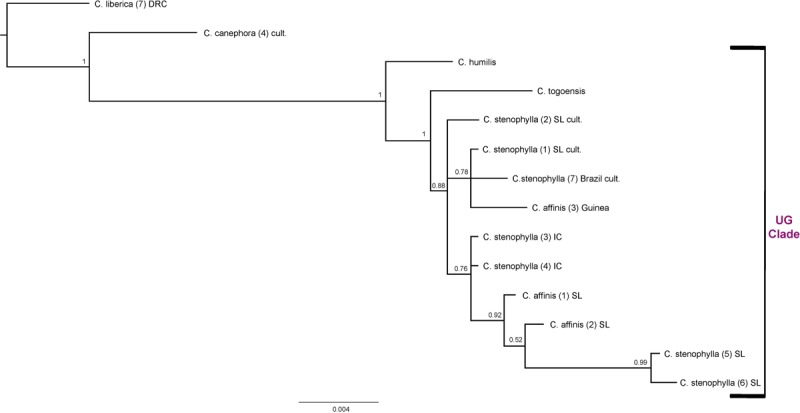
Combined ITS and plastid maximum clade credibility tree. Bayesian posterior probabilities are indicated above branches. See Tables 1, 2 for accession information. Country abbreviations: IC, Ivory Coast; SL, Sierra Leone. Clade terminology follows Maurin et al. (2007) and Davis et al. (2011): UG, Upper Guinea.

## Discussion

### Historical and Present-Day Status of *C. affinis* and *C. stenophylla*

In 2018, we rediscovered *C. stenophylla* in two locations in Sierra Leone, one from where it been collected before (Kasewe Hills, in 1954) and one a new location (Kambui Hills) (Figure 3). In both locations, *C. stenophylla* is extremely localized, and seemingly threatened. In the Kasewe Hills, near Moyamba, we were only able to locate a single plant, in an area of high deforestation. In the Kambui Hills, near Kenema, we located a small population, the extent of which is as yet unknown, but there are ongoing threats from logging, human encroachment, and artisanal gold mining. In late 2019, we located *C. affinis* in the Kambui Hills, not far from the populations of *C. stenophylla*. This is the first record of this species from the wild in Sierra Leone. The present day status of *C. stenophylla* and *C. affinis* in Guinea and Ivory Coast is poorly known. In Guinea, *C. stenophylla* was recorded as being under limited small scale cultivation in the 1980s (Le Pierrès et al., 1989), but there were no records of wild plants at that time. From a basic survey of remaining forest cover in Guinea, using satellite imagery (Google Earth, 2019), the likelihood of finding *C. affinis* and *C. stenophylla* in many of the localities where it was previously recorded as an indigenous plant (see the section “Results”; Figure 3) is limited, although possible. Field survey in more remote locations in Guinea, in appropriate environments and elevations, may reveal wild populations of these species. Indeed, we report here on a recent collection [2015; *Couch 757* (K)] of a sterile (no flowers or fruit) coffee specimen from Guinea, which was identified on the basis of our DNA sequencing and morphology as *C. affinis* (see below). Despite this encouraging find, deforestation rates in Guinea are very high and ongoing (Couch et al., 2019); in 1992 it was calculated that 96% of the original forest had already been destroyed (Sayer et al., 1992). In Ivory Coast, the likelihood of finding more extensive wild populations of *C. stenophylla* and *C. affinis* are better, particularly as satellite data (Google Earth, 2019) shows the existence of native remnant vegetation in localities where this species was previously recorded, and where it is likely to be located. That said, one of the best known forest sites for this species in Ivory Coast (Berthaud, 1986), i.e., at Ira Forest (Forêt L’Ira), was largely destroyed around 2008. In summary, *C. stenophylla* and *C. affinis* are threatened throughout their indigenous ranges, and particularly in Guinea. On the IUCN Red List *C. stenophylla* is assessed as Vulnerable (VU); *C. affinis* is currently Data Deficient (DD) (International Union for Conservation of Nature (IUCN), 2020). On the basis of our literature, herbarium, and field survey, and DNA analysis, *C. stenophylla* and *C. affinis* are indigenous species of Guinea, Ivory Coast and Sierra Leone (Figure 3).

Historical data shows that *C. stenophylla* and *C. affinis* were farmed in some quantity in Upper West Africa, and especially *C. stenophylla* in Guinea and Sierra Leone. *Coffea stenophylla* was also widely cultivated in research stations across Africa and in various Asian countries; the presence of *C. affinis* in research stations appears to have been restricted to Upper West Africa (Guinea, Ivory Coast) during the early part of the last century. Our field surveys in Sierra Leone indicates that neither *C. stenophylla* nor *C. affinis* are under commercial cultivation (i.e., being farmed), or otherwise cultivated, in the present day. The last confirmed record of *C. stenophylla* production in Sierra Leone may have been the small plantation at Njala University grounds, which was apparently cut down after being abandoned in the 1980s (A. Lebbie pers. comm.). In Guinea, *C. stenophylla* was recorded as being under limited small scale cultivation in the 1980s (Le Pierrès et al., 1989). *Coffea stenophylla* has been recorded recently in several coffee research collections including the Centre National de Recherche Agronomique (CNRA), in Ivory Coast (Figure 2A); L’Institut de recherche pour le développement (IRD), France [A. Davis pers. observ.], and Entebbe Botanical Gardens, Uganda [A. Davis pers. observ.]. It is also likely to exist in other *ex situ* collections. However, across the *ex situ* coffee germplasm network there is the problem of duplication, i.e., the same genotype(s) being represented in multiple sites (Anthony et al., 2007; Bramel et al., 2017; Davis et al., 2019). There also seems to be some confusion with the narrow-leaved variant of *C. arabica* ‘Angustifolia,’ which owing to its narrow leaves is sometimes mistakenly accessioned as *C. stenophylla*. *Coffea affinis* has not been recorded in coffee research collections since the beginning of the twentieth century. Genotyping by sequencing, or similar methods, are required to make a full assessment of *ex situ* collections of *C. stenophylla*, and all other coffee species.

### Natural Habitat (Growing Environment) of *C. affinis* and *C. stenophylla*

Knowing the location and associated habitat of crop wild relatives is important, as it can provide an initial assessment of environmental suitability as a crop plant. Our literature survey indicates that *C. stenophylla* may have drought tolerance characteristics (Portères, 1937b; Wellman, 1961; Wrigley, 1988). Both species occur at relatively low elevations (150–700 m) (De Wildeman, 1906a; Watt, 1908; Portères, 1937b). In Ivory Coast (at Ira Forest) *C. stenophylla* occurs on the upper, drier parts of hills; in the same location, *C. canephora* and *C. liberica* was found in the valleys (i.e., the lower, wetter areas). The locations for this species in Ivory Coast are generally drier than Sierra Leone (see below), with rainfall in the region of 1,500–1,700 mm per year, a 3–4 months dry season (Portères, 1937b), and an average annual temperature of c. 25.5°C. In Guinea, De Wildeman (1906a) reported that in its natural state *C. affinis* occurs in gallery forest (forest associated with rivers) bordering waterfalls and in humid (evergreen) forest, and that it was frequently found at elevations of 400–700 m at a distance of 100 to 300 km from the sea.

In Sierra Leone our fieldwork located *C. stenophylla* at precisely 400 m at two locations (Kasewe and Kambui Hills), even when there was sufficient forest down to 200 m. We visited higher elevation locations within the Western Peninsula National Park (up to 600 m), but did not locate either of the two species. Sierra Leone is generally not a mountainous country, and most of its land is below 500 m. At Kasewe and Kambui Hills the climate is tropical monsoonal, with an annual rainfall average of 2,350–2,650 mm, an average annual temperature of c. 26°C, and a distinct 3–4 months dry season (November to March/April). It should be noted that there is a considerable difference in rainfall between the wild locations of *C. affinis* and *C. stenophylla* in Ivory Coast and Sierra Leone, although this requires careful verification.

Our herbarium survey did not provide a great deal of further information on the habitat of either of these two species. Notes on herbarium specimens for *C. stenophylla* infrequently state ‘hills,’ one specimen states ‘very common [on] more open places on augite hills,’ and another ‘dans les montagnes de Sierra Leone,’ which suggested an association with high ground topology.

### Species Status and Systematic Affinities of *C. affinis* and *C. stenophylla*

A recent hybrid origin for *C. affinis*, as a result of crossing between *C. liberica* and *C. stenophylla* (Chevalier, 1929; Cramer, 1957; Stoffelen, 1998) is ruled out on three counts: (1) *C. affinis* is clearly fertile and productive, as evidenced from literature records (Portères, 1937a), herbarium specimens (with plentiful seed), and recent (2020) field observation (D. Sarmu pers. observ.; Figure 2C). Diploid interspecies hybrids of coffee are usually sterile, and while they may produce flowers, fruit set and production of viable seed are minimal unless fertility is restored via polyploidization (usually tetraploids) (Carvalho and Monaco, 1968; Charrier, 1978). (2) The fruits of *C. affinis* are always described as black; the hybrid *C. liberica* × *C. stenophylla* (see below) has purple fruits. (3) Our samples of *C. affinis* do not alternate between ITS and plastid markers, as in known hybrids [*C. arabica*, *C. arabica* × *C. racemosa*, *C. liberica* × *C. eugenioides*, and *C. liberica* × *C. stenophylla* (see below)], but instead are consistently resolved as sister to *C. stenophylla*. The idea that *C. affinis* is a fixed mutation of *C. stenophylla*, (Wellman, 1961) is also ruled out because of the variation evident in this taxon.

Our DNA analyses infer that *C. stenophylla* and *C. affinis* are closely related. Separate ITS (Figure 4) and plastid analysis (Figure 5) fail to resolve the systematic positions of the four Upper Guinea (UG) clade species (i.e., including *C. humilis* and *C. togoensis*), but a combined analysis of these data sets retrieves monophyly monophyly for *C. affinis* and *C. stenophylla* (Figure 6). *Coffea affinis* and *C. stenophylla* share specific characters, including: the habit of a small tree, obovate leaves with a distinct apical tip (acumen) and drying green, 2 to 4 flowers per axil, 6- to 8-merous flowers (i.e., six to eight corolla lobes and anthers per flower; and black or black–purple fruits (see Figure 2). *Coffea togoensis*, from Ghana and Togo, is also a small tree, and has leaves with a distinct apical tip (acumen), 6- to 8-merous flowers and black fruits, but generally has elliptic leaves, drying grayish or gray-green, 1 or 2 flowers per axil, and smaller fruits and seeds. *Coffea humilis* is unlike *C. affinis*, *C. stenophylla* and *C. togoensis*, as it is a monocaul dwarf (single-stemmed woody plant, up to 1 m high), with large (up to 22 cm long) obovate leaves, 5- to 7-merous flowers and red fruits.

*Coffea stenophylla* and *C. affinis* exhibit considerable morphological variation, particularly with regard to leaf size and shape. Some examples of *C. stenophylla* approach *C. affinis* in terms of leaf shape and dimensions. Further work, including morphological and more detailed molecular study, is required to determine the precise relationship between these two species. There could be grounds for subsuming *C. affinis* within *C. stenophylla*, for example as *C. stenophylla* var. *camaya* (Portères, 1937a).

Our historical accession of farmed *C. affinis* from a coffee plantation in Sierra Leone (two genotypes from a single accession [Cope *s.n.*, 7 iii 1912 (K)] is not related to either *C. affinis* or *C. stenophylla*. ITS and plastid marker data place this accession in the Lower Guinea/Congolian Clade. The specimens of this accession are a reasonable morphological match for *C. affinis*, with leaves of the same dimensions and possessing an acuminate leaf tip, but the seeds are somewhat larger and narrower than *C. affinis*; flower morphology and fruits color are unknown. The Cope *s.n.* accession is compelling, as it placed with the Liberica Alliance but does not conform to any of the known species in this alliance. The distinct, elongated leaf tip (acumen) immediately sets it apart from all variants of *C. liberica* (Stoffelen, 1998).

In both ITS (Figure 4) and plastid (Figure 5) analyses *C. liberica* is not monophyletic. Further molecular data is required for *C. liberica* and closely related taxa. As currently circumscribed, *C. liberica* is a highly polymorphic (Bridson, 1985, 1988; Stoffelen, 1998; Davis et al., 2006) encompassing a broad range of morphological variation.

### Identification of *Coffea* Species Hybrids

Identification of interspecies hybrids or introgressed plants on the basis of incongruence between biparental (e.g., nuclear) and uniparental (plastid) phylogenetic trees is well established (Linder and Rieseberg, 2004). In most flowering plants plastid DNA is maternally inherited, whereas the nuclear DNA and the ITS region of ribosomal DNA is not (Chase et al., 2005). Previous analysis of ITS and plastid markers in *Coffea* have been used to identify the parents of the allotetraploid *C. arabica* (*C. eugenioides* × *C. canephora*) and the diploid hybrid *C. eugenioides* × *C. liberica* (Maurin et al., 2007). As part of this study, we undertook further tests of this method using an additional interspecies hybrids of known crossing history, viz. *C. arabica* × *C. racemosa* (Medina Filho et al., 1977a, b), which demonstrated the utility of the method, at least for recently produced hybrids (Figures 4, 5). Using this method, we were able to identify the interspecies hybrid *C. liberica* × *C. stenophylla* (Figures 4, 5) from cultivated material collected by us in Sierra Leone (see Table 1). The hybrid *C. liberica* × *C. stenophylla* has been reported before (Cramer, 1957), but never authenticated. The accession of this hybrid from Sierra Leone appears to be partially or totally sterile; in some years it produces a few fruits, but neither the viability nor fertility of seeds have been tested. The low level of fruit setting suggests that this hybrid is a diploid (2*n* = 22) rather than a polyploid; tetraploids generally have higher fertility or restored fertility (Carvalho and Monaco, 1968; Charrier, 1978). We have no means of knowing whether this hybrid was the result of a cross in cultivation or in the wild. Either origin is plausible: both species were grown together in a number of research stations in Africa, and Asia (Cramer, 1957); and *in situ* crossings have been reported. In Ivory Coast hybrid seedlings (but not mature plants) of *C. liberica* × *C. stenophylla*, were detected in the wild (Berthaud, 1986). Generally, interspecific hybridization in natural populations of *Coffea* is a rare phenomenon (Charrier, 1978; Berthaud, 1986).

Interspecies hybrids, once fertility (and thus yield) is restored via conversion to the tetraploid (4*n* = 44) state (Carvalho and Monaco, 1968; Nagai et al., 2008), are valuable for coffee crop development, as they provide the possibility of introducing useful traits. For example, CLR resistance (*C. canephora* × *C. arabica*; Clarindo et al., 2013; Avelino et al., 2015) and leaf-miner resistance (*C. arabica* × *C. racemosa*; Medina Filho et al., 1977b) for Arabica coffee. In our DNA survey of long-styled African species [Coffee Crop Wild Relative Priority Groups I and II (Davis et al., 2019)] we confirm that hybridization is possible across all the major African coffee clades (lineages), indicating the potential to create custom interspecies hybrids across this wide spectrum of *Coffea* species diversity.

## Conclusion

*Coffea affinis* and *C. stenophylla* may possess useful attributes for coffee crop plant development, including taste, disease resistance, and climate resilience. These attributes would be best accessed via breeding programs, including those involving interspecies crossing, followed by tetraploidization. Here, we confirm that (initial) hybridization is possible across all the major clades of long-styled African *Coffea* species (Maurin et al., 2007; Davis et al., 2011; Hamon et al., 2017), i.e., Coffee Crop Wild Relative (Priority) Groups I and II (Davis et al., 2019). For *C. stenophylla* we confirm via DNA sequencing that a cross can be made with *C. liberica*, supporting the work of Louarn (1992), who also demonstrated that *C. stenophylla* can be crossed with *C. canephora*, *C. congensis*, and *C. pseudozanguebariae*. Development of *C. stenophylla* and *C. affinis* via minimal domestication (e.g., the selection of trait-specific genotypes) may be possible, although this route would probably only be feasible for high-value markets, such as the upper end of the speciality coffee sector, based on the historical reports of its superior taste. Productivity (green coffee yield) appears to be lower than the major commercial species (*C. arabica*, *C. canephora* and *C. liberica*). A key caveat here, is that *C. stenophylla* has not undergone sensory or agronomic evaluation in a contemporary setting. Despite the shortfall in our understanding of these species, the available evidence, as summarized and reviewed here, is more than sufficient to warrant further research for *C. affinis* and *C. stenophylla*, and to take measures to ensure their survival in the wild (*in situ*) and in cultivation (*ex situ*). Deforestation, and other forms of land-use change, are threatening the survival of these species in the wild, in Sierra Leone, Ivory Coast and Guinea.

The decline in the use of *C. stenophylla* use as a crop plant in Upper West Africa was dramatic, from once widespread (although comparatively small scale) use in the late and early parts of the 19th and 20th centuries, to apparently nothing today. Our survey of local farming communities in Sierra Leone, reported an absence of indigenous knowledge for this species. One of the other main reasons for the decline in its use may have been the considerable agronomic and commercial success of robusta coffee (*C. canephora*), which was introduced into global cultivation around the same time as *C. stenophylla* and greatly surpassed the comparatively meagre productivity of other underutilized coffee species (Davis et al., 2019). Following on from our fieldwork in Sierra Leone (2018–2020), wild stock of *C. affinis* and *C. stenophylla* is now being propagated in quantity, for sensory and agronomic evaluation, and to safeguard its existence. Field work in Guinea and Ivory Coast are required to further ascertain the present day indigenous and cultivated (farmed) status, respectively, and to provide a conservation management plan to ensure its survival in the wild. An in-depth review of coffee research collections, including genotyping (genome banking), is required to formulate an effective *ex situ* conservation management strategy for *C. affinis* and *C. stenophylla*, and indeed many other coffee species. African coffee species provide key resources for the sustainability of the global coffee sector (Davis et al., 2019) and should receive appropriate conservation measures in the wild (*in situ*) and in cultivation (*ex situ*).

## Data Availability Statement

The accession numbers for the sequencing data presented in this article can be found in Table 2 within the article.

## Author Contributions

AD devised the non-fieldwork elements of the study, undertook the literature and herbarium, participated in fieldwork, and was the lead on writing the manuscript. RG undertook the DNA sequencing and analyses, and contributed toward the writing of the manuscript. MF helped to devise the study, provided assistance with DNA sequencing, and contributed toward the writing of the manuscript. DS undertook the bulk of the fieldwork in Sierra Leone. JH devised the overall framework of the study, devised and undertook substantial fieldwork in Sierra Leone, and contributed toward the writing of the manuscript.

## Conflict of Interest

The authors declare that the research was conducted in the absence of any commercial or financial relationships that could be construed as a potential conflict of interest.
